# Promoting male involvement to improve PMTCT uptake and reduce antenatal HIV infection: a cluster randomized controlled trial protocol

**DOI:** 10.1186/1471-2458-11-778

**Published:** 2011-10-10

**Authors:** Karl Peltzer, Deborah Jones, Stephen M Weiss, Elisa Shikwane

**Affiliations:** 1 HIV/AIDS/STI and TB (HAST) Research Programme, Human Sciences Research Council, Pretoria, South Africa; 2 Department of Psychology, University of the Free State, Bloemfontain, South Africa; 3 Department of Psychiatry and Behavioural Sciences, University of Miami Miller School of Medicine, Miami, Florida, USA

## Abstract

**Background:**

Despite the availability of a dual therapy treatment protocol and infant feeding guidelines designed to prevent mother to child transmission (PMTCT) of HIV, of the over 1 million babies born in South Africa each year, only 70% of those born to HIV positive mothers receive dual therapy. Similar to other resource-poor nations facing the integration of PMTCT into routine pregnancy and infant care, efforts in South Africa to scale up PMTCT and reduce transmission to < 5% have fallen far short of the United Nation's goal of 50% reductions in paediatric HIV by 80% coverage of mothers.

**Methods/Design:**

This study proposes to evaluate the impact of combining two evidence-based interventions: a couple's risk reduction intervention with an evidence based medication adherence intervention to enhance male participation in combination with improving medication and PMTCT adherence in antenatal clinics to increase PMTCT overall reach and effectiveness. The study will use a group-randomized design, recruiting 240 couples from 12 clinics. Clinics will be randomly assigned to experimental and control conditions and effectiveness of the combined intervention to enhance PMTCT as well as reduce antenatal seroconversion by both individuals and clinics will be examined.

**Discussion:**

Shared intervention elements may decrease sexual risk and enhance PMTCT uptake, e.g., increased male participation, enhanced communication, HIV counselling and testing, adherence, serostatus disclosure, suggest that a combined sexual risk reduction and adherence intervention plus PMTCT can increase male participation, increase couples' communication and encourage adherence to the PMTCT process. The findings will impact public health and will enable the health ministry to formulate policy related to male involvement in PMTCT, which will result in PMTCT.

**Trial registration:**

PACTR201109000318329

## Background

Despite the availability of a dual therapy treatment protocol (Nevirapine + AZT) and infant feeding guidelines designed to prevent mother to child transmission (PMTCT) of HIV, of the over 1 million babies born in South Africa each year, only 70% of those born to HIV positive mothers receive dual therapy [1]. Similar to other resource-poor nations facing the integration of PMTCT into routine pregnancy and infant care, efforts in South Africa to scale up PMTCT and reduce transmission to < 5% have fallen far short of the UN goal of 50% reductions in paediatric HIV by 80% coverage of mothers [2]. Bringing transmission rates below 5% represents potentially saving 75,000 babies of the 300,000 exposed to HIV annually. Mpumulanga Province, the focus of the current application, has consistently had one of the lowest rates of PMTCT; 69% of pregnant women received PMTCT services in 2009. Despite increases in uptake in other regions [3] Mpumalanga has the highest HIV prevalence (4.5%) among children (0-18 years) in South Africa [4]. PMTCT program failure occurs at all stages of the process in South Africa [5]. Implementation of PMTCT programs in already overburdened clinical settings presents multiple challenges, including systemic (e.g., failure to offer ARV prophylaxis, home delivery), social (e.g., stigma, lack of disclosure), individual (e.g., maternal failure to ingest medication or provide it to the infant, failure to obtain antenatal testing) and interpersonal (e.g., lack of male involvement, intimate partner violence) factors. Increasing male participation as a method to enhance implementation of PMTCT and increase uptake of and commitment to the medical protocol for pregnancy and newborn care has been identified as a potentially critical strategy for PEPFAR countries [6,7]. This application proposes an implementation strategy to test whether male involvement will increase PMTCT uptake utilizing the existing public health program linking antenatal HIV Counselling and Testing (HCT) and PMTCT services.

Male involvement is also essential due to a related issue, the disturbingly high rate of HIV seroconversion (3%) during pregnancy in South Africa [8]. This suggests the continuation of unprotected high risk sex during the middle to latter stages of pregnancy may go undetected for the purposes of PMTCT unless women are re-tested just prior to delivery [9]. Again, male involvement in the antenatal/HCT process may also influence and reduce risk of HIV exposure during this critical period. Prevention of Mother to Child Transmission (PMTCT) has played the major role in reducing child mortality associated with HIV/AIDS and improving maternal health [10,11]. Guidelines for the use of antiretroviral therapies to reduce transmission have been implemented in the most HIV affected countries, especially those in sub-Saharan Africa [12]. The PMTCT program in South Africa consists of comprehensive counselling, HIV-testing and the offer of ARV prophylaxis for seropositive mothers and their newborns and referral of HIV positive mothers and their families for CD4 count assessment for antiretroviral therapy [10]. However, despite the widespread availability of PMTCT, not all mothers provided with medication take it themselves or provide it to their newborns [13-17] due to a variety of circumstances, including unwillingness or perceived inability to disclose their HIV status to their partners [18]. Mother to child transmission rates range from 12% to > 20% in South Africa and drop out is high at all stages of the PMTCT process [1].

To date, HCT for pregnant women has largely been organized on an individual and sex-specific basis in PMTCT programs, typically ignored by male partners. However, a couples approach to HCT and antenatal care facilitates communication about HIV serostatus, thereby reducing one of the major barriers to acceptance of ARV prophylaxis by mothers for themselves and their newborns, as well as encouraging adoption of preventive behaviours within couples to reduce HIV incidence during pregnancy [19] The need for male involvement in the PMTCT process has been increasingly encouraged to improve adherence to ARV prophylaxis [20,21] though no randomized clinical trials of the influence of male partners as key contributors to acceptance and PMTCT uptake have been conducted. HCT and prevention strategies for couples in stable relationships could also strengthen HIV prevention efforts [22] in Southern Africa, where the majority of HIV infections occur in stable relationships. Prevention programs to increase male involvement in Tanzania, Botswana and Zambia have met with some success, e.g., Tanzania found male involvement increased NVP uptake [23], Botswana utilized a media campaign and increased male involvement from 4% to 11%, and Zambia utilized monetary incentives and couples counselling and increased male involvement in PMTCT [24]. In Cote d'Ivoire, prenatal couple counselling and testing improved couples' communication on sexual risks among both HIV positive and negative women [25]. In Kenya, partner participation in HCT and couples counselling increased Nevirapine and formula feeding uptake among women attending antenatal clinics [26] and partner attendance to 15% [27,28]. In Rwanda and Zambia, couples HCT led to enhanced follow up among pregnant women at both sites but did not increase Nevirapine uptake [29]. However, while PMTCT attendance by both members of a couple is feasible [23], uptake remains limited by lack of male participation [29] highlighting the need to increase communication within the couple about reproduction and sexual health [30]

Men's attitudes regarding involvement in PMTCT and antenatal care (ANC) programs have been linked to cultural barriers, including the perception that male participation in ANC/PMTCT services is superfluous and that ANC is "a woman's responsibility" [30,10]. Additionally, men in Tanzania were found to have general HIV knowledge but to lack specific information regarding PMTCT and unable to attend PMTCT programs due to timings which conflict with their work schedules. Similarly, in Zambia, men were perceived as decision makers in the home and felt their position was undermined if they were expected to attend a "women's clinic program", leading them to decline to attend ANC and PMTCT with their partners [24], with as few as 2% attending urban ANC (personal communication, Provincial Health Office, Lusaka, Zambia). In Botswana, men regarded ANC health facilities as being "generally unfriendly" to them. Most recently, a review of studies incorporating men suggested that male "support" as well as "involvement" is key to increasing PMTCT uptake [31]

As noted, 3% of women in South Africa seroconvert during pregnancy following receipt of HIV negative test results [9,8]. Following diagnosis, partners may not disclose their serostatus [32] or protect an uninfected partner [33] These data highlight the urgent need to incorporate strategies in conjunction with the PMTCT process to prevent HIV transmission to mothers during pregnancy [34,35]. This team's previous research in the US and Zambia has found that a gender specific group, sexual behaviour intervention designed to increase couples communication, *the Partner Project*, enhanced the acceptability and use of sexual barrier products (male and female condoms) among HIV seropositive men [36] and women [37,38]. We also increased need for multiple session interventions among those in serodiscordant relationships, who appear to represent a unique population within those couples living with HIV [39]. The Partner Project, currently being implemented in community health clinics across Zambia by CDC Zambia, has achieved 95-100% retention of enrolled couples over 6 months and 90-95% over 12 months and maintained comparative levels of participation by both men and women throughout the intervention.

### Aim of the study

The aim of the study is to evaluate the impact of combining two evidence-based interventions: a couple's risk reduction intervention with an evidence based medication adherence intervention to enhance male participation in combination with improving medication and PMTCT adherence in antenatal clinics (ANCs) to increase PMTCT overall reach and effectiveness. The study will use a group-randomized design, recruiting 240 couples from 12 clinics. Clinics will be randomly assigned to experimental and control conditions and effectiveness of the combined intervention to enhance PMTCT as well as reduce antenatal seroconversion by both individuals and clinics will be examined.

#### Objectives

Study objectives are to enhance PMTCT effectiveness by 1) increasing male partner participation in the PMTCT process, 2) increasing male HCT, 3) increasing maternal and infant adherence to the overall PMTCT protocol and 4) increasing the use of sexual barriers and reducing HIV transmission to mothers during pregnancy.

## Methods/Design

### Setting

Gert Sibande District (pop. 890,699, 71 PMTCT sites) and Nkangala District (pop. 1,020,592, 80 PMTCT sites) in Mpumalanga Province, continue to show evidence of an increase in antenatal HIV prevalence from 32.1% in 2006 to 34.6% in 2007 to 35.5% in 2008 [2]. Gert Sibande district had the highest antenatal HIV prevalence (38.9%) in Mpumalanga; 52% of those mothers identified as positive took Nevirapine to prevent vertical HIV transmission in 2006 [40]. HCT uptake overall is 70% in Mpumalanga province and below 50% in Gert Sibande District. The delivery rate in health facilities is approximately 70%, well below the South African average [41]. Clinic sites will be comparable by South African criteria for PMTCT sites: on-site counselling and testing for HIV & private room for HCT; daily availability of HCT; referral to ARV site & CD4 count testing; ARV prophylaxis; antenatal counselling on infant feeding; postnatal counselling and support for infant feeding & free infant formula; PCR testing for infants for HIV; at least two trained PMTCT staff and two lay counsellors; and a support group specific to HIV-positive mothers and pregnant women.

### Design

**Figure 1:**
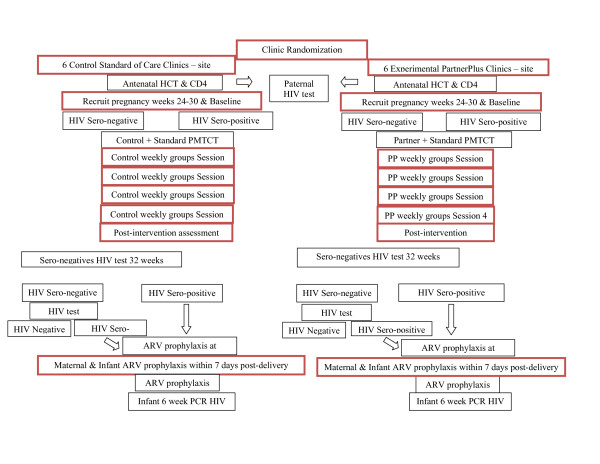
**PartnerPlus Study Flowchart**.

This study is a group-randomized controlled trial using a 2 × 6 comparison (Clinic, Experimental, Control × Time, Baseline, Post-Intervention, Pre-delivery 32 weeks, Pre-delivery day, Delivery, Post Partum). Twelve community health centres in communities within the Gert Sibande and Nkangala Districts in Mpumalanga, South Africa will be randomly assigned to condition in a 1:1 ratio. Six usual care condition clinics will provide the standard of care, PMTCT; six experimental condition clinics will offer PartnerPlus to mothers completing HIV testing, regardless of serostatus, who are willing to enrol with their male partners and participate in the integrated PartnerPlus intervention. This study will recruit 240 couples (n = 480 individuals); community clinics will recruit 2 cohort per clinic over 3 months (10 couples per cohort, n = 40, 120 individuals per condition) (see Figure 1).

### Study hypotheses

#### Hypothesis 1

Community clinics implementing PartnerPlus will have more effective PMTCT program adherence, as measured by maternal and paternal ANC and PartnerPlus visits, maternal and paternal HCT uptake, maternal and infant Nevirapine and AZT dose uptake, infant PCR, maternal and infant HIV serostatus.

#### Hypothesis 1a

Community clinics implementing PartnerPlus will have reduced sexual risk behaviour, as measured by participant sexual barrier use.

### Principles for recruitment

#### Inclusion criteria

***Community health centres ***with a high antenatal care clinic case-load (based on statistics collected by the Department of Health) in each of the two study districts with a high burden of antenatal HIV will be included in the study.

#### Inclusion criteria

Eligible participants will be male and female. Women: a) pregnant women age 18 and older who have received HCT at the ANC, b) willing to attend PartnerPlus or PMTCT visits with their male partners, c) male partners must also be available to participate and d) both partners must be willing to participate in the study. Men: a) men aged 18 and older who are partners of pregnant women enrolled in the study, b) willing to attend PartnerPlus or PMTCT visits with their female partners, c) female partners must also be enrolled in the study and available to participate. All female participants must have received HIV counselling and testing. In the event that a participant has refused to know their status, they will continue to be eligible as long as the results of testing are provided to the study through clinic records. All participants will be current adult residents of Mpumalanga Province and agree to attend four group sessions, 2 psychosocial assessments and 1 within 7 days post-delivery assessment (maternal and infant only - blood sample assessment).

#### Exclusion criteria

Women who are not currently pregnant or are unwilling to receive HCT are not eligible for this study. Women whose partners are not willing to participate are not eligible for this study. Men whose pregnant partners are unwilling to participate are not eligible for this study. Women and men who are not couples are not eligible to participate. Participants unable to provide informed consent will not be eligible.

#### Randomization

Clinics will be randomly assigned as PartnerPlus sites or standard of care sites, stratified by antenatal care clinic case load. Randomization will be conducted using a secure remote randomization service. Each of the 2 communities will have six community health centre sites participating in the study. PartnerPlus community health centres will offer the PartnerPlus (Partner plus PMTCT standard of care); standard of care community health centres will offer the standard of care, PMTCT, and a time matched session. The time matched group session will consist of a video on health education issues. Participants in both conditions will receive time matched interventions and identical compensation. Each clinic will recruit a total of 1 cohort during a 3 month period.

#### Blinding

Participants (clinic staff members and clinic patients) will not be blind to their intervention status. However, to protect against information biases in reporting sexual behaviour and ARV adherence behaviour, the data collection team who will assess the outcomes will be blind to the clinic's status as intervention or control intervention arm.

### Procedure

Men and women in the local community will be recruited to work as "Peer Mentors" (men) and "Lay Counsellors" (women) in the project. Recruitment of Peer Mentors and Lay Counsellors will be done with the assistance of nurses at the clinics, community leaders, and HIV/AIDS coordinators at the district level, who will be briefed on the objectives of the project. After recruitment, Mentors and Counsellors will be trained over five days on PMTCT, gender issues and facilitation skills and 10 days in HCT, and will participate as group leaders.

Participants (N = 240 couples) will be recruited in 2 communities through six community health clinics in each community. At the ANC, following VCT, participants will be informed about the study and referred for participation if interested. Following initial contact with prospective participants, study staff will obtain a medical and social (couple) history to determine initial eligibility for the study. Couples will complete a couples status verification questionnaire [42] Couples are interviewed separately for couple eligibility, and are asked a short series of rotating questions related to couple-specific issues e.g., "who sleeps next to the window? What colour is your blanket?" Staff will compare responses to access couple responses and qualify or disqualify couples at this point. Couples are not notified of the reason for ineligibility but are advised that they have not met eligibility requirements, and are compensated for the visit and thanked for their interest in the study. Those meeting basic study criteria will be invited to provide and sign the Informed Consent form. Participants will then complete a baseline psychosocial and behavioural assessment and be scheduled for their next visit. All questionnaire data are obtained by being read to the participants to eliminate potential confounds related to literacy.

#### Consent

As participants can choose to participate in the intervention or continue to receive the standard of care, it is not necessary to consent the entire ANC patient population in each clinic. Only women who have completed HCT will be approached to participate in the study with their male partners. Informed consent will be obtained which will include permission to give the contact details to the research staff, access to medical file information, participate in the experimental or control condition, tape recording of intervention sessions and psychosocial interview and blood specimen collection assessment procedures. To ensure comprehension of both literate and illiterate participants, the informed consent will be reviewed in detail (actually read to the prospective participant in their primary language) and each study candidate will be asked to sign the form after agreeing to its terms. Witnesses to consent will be drawn from study staff. Participants will have been briefed on the nature of the study prior to referral for informed consent.

#### Interventions

##### Standard of Care and health education videos

According to the South African guidelines for standard of care all pregnant women should be routinely offered HIV counselling and testing and encouraged to have partner or spouse testing.

HIV seronegative. Following a negative HIV test, women should have post-test counselling and counselling on risk reduction interventions including involvement of partners or spouses, focusing mainly on how to maintain their HIV-negative status. They should continue to receive routine antenatal care, and should be encouraged to use condoms. They should be offered a repeat HIV test at or around 32 weeks gestation, to detect those who may have sero-converted during pregnancy.

HIV seropositive. Following a seropositive HIV test, pregnant women should be assessed for CD4 at the laboratory. Women are routinely counselled on safer sex, family planning, postnatal contraception and partner testing. Women are provided with ARV regimens for PMTCT. This is followed by a second visit, at which time the clinic Physician may prescribe ARVs if WHO guidelines for ARV introduction are met (CD4 < 350). HIV seropositive patients are monitored by the healthcare worker at the antenatal clinic until 6 weeks post-partum, at which time they are referred to the appropriate facility.

Study participants, women and men, will view educational videos currently being used in the community that address healthy living issues (e.g., nutrition, exercise, relaxation). Participants in Usual Care will receive the PMTCT protocol plus the time matched group administered presentation of health education videos.

#### Cognitive Behavioural Risk Reduction (CBRR) Group Counselling Intervention

The Group intervention employs a closed, structured, group intervention limited to 10 participants (women or men). Four weekly, 11/2-2 hour sessions are led by a trained gender-congruent counsellor and a peer facilitator and emphasize group participation, cognitive-behavioural skill building, sexual negotiation and experimentation with products. The impact of alcohol and drug use as direct and indirect contributors to high risk behaviours is addressed. Participants receive cognitive behavioural skill training on HIV/STD prevention and transmission, risk reduction strategies including circumcision and reproductive choice issues, communication, conflict resolution, sexual negotiation and an educational/experiential program to increase use of and adherence to sexual barriers. Information is presented through multiple modalities (i.e., visual, auditory, experiential) with ample opportunities for practice, feedback, and reinforcement (e.g., discussion of methods of circumcision, reproductive choice, sharing experiences using products with their partners, questions on product use, opportunity to handle and examine products). Participants are introduced to sexual barrier products (male and female condoms) for the purpose of determining cultural acceptability of such products to increase their uptake. The content of the women's and men's interventions include gender-relevant issues (e.g., relationships and sexual negotiation), self management techniques (e.g., learning to recognize antecedents of conflict and to control impulses to engage in maladaptive and unproductive coping responses) and assertiveness training (e.g., role plays to teach appropriate communication strategies).

The intervention is guided by the theories of reasoned action (intentions influence attitudes and subjective norms which influence beliefs about behaviour; [43] and planned behaviour (perceived behavioural control influences intentions and behaviour; [44] as predictors of sexual barrier use [45]. Within this model, it is hypothesized that sexual behavioural intentions and HIV-related knowledge influence attitudes and perceived norms regarding barrier use and partners, maladaptive coping strategies, substance use and medication adherence. Perceived sexual self efficacy, control of sexual behaviour and barrier use will influence risk reduction strategies and future sexual behaviour.

*Session One *addresses HIV/Sexually Transmitted Infections (STIs), safer sex, barrier use, reproductive choice and communication. Participants are advised of standard rules regarding within-group confidentiality for both participants and staff, stressing the restriction on information overlap between gender groups. The use of gender concordant therapists reduces the risk of disclosure of group information. The HIV/STI and safer sex segment informs the participants about the need for safer sex regardless of serostatus (HIV transmission, infection with HIV/STIs) and the health implications for participants and their partners. This segment includes a discussion of the hierarchical method of sexual barrier use [46,47]. Male and female condoms are introduced as the most effective forms of sexual protection. This is followed by "hands-on" demonstrations of both products, including practice with placing male and female condoms on/in models. The female condom is also illustrated with anatomical charts to clarify procedures for insertion. All available options for protecting against HIV infection are discussed in this session. For example, male circumcision will be noted as one means of reducing risk of HIV infection. Cognitive/behavioural skill training and communication skills in relationships [48] are introduced in the context of awareness building and cognitive reframing, heightening participants' awareness of their reactions to barrier use in their sexual relationships and reframing automatic thoughts that may impede barrier use and communication [49]. Participants will learn self management techniques (e.g., learning to recognize antecedents of conflict). At the conclusion of the session, participants complete a confidential sexual diary to record their sexual behaviour and use of barriers. Sexual diary entries are not discussed during groups. Participants receive and are encouraged to use a week's supply of male and female condoms; handouts are provided on male and female condoms and reproductive choice.

*Session Two *begins with participants' completion of sexual diaries and a questionnaire assessing the acceptability of male and female condoms. The session follows a similar format to Session One Cognitive/behavioural skill building focuses on sexual negotiation and open communication techniques in relationships and the influences of family. At the end of the session, participants receive a week's supply of male and female condoms.

*Session Three *begins with participants' completion of sexual diaries and a questionnaire assessing the acceptability of the products. Participants are encouraged to discuss their experiences with the products, and the reactions of partners and problems encountered. The group process facilitates the sharing of experiences by those who have tried the products to encourage those who have not. The session addresses the potential for engaging in high risk sexual behaviour when under the influence of alcohol and/or drugs. Skill building focuses on sexual negotiation, influencing [50] and positive communication (e.g., expressing appreciation, avoiding blaming and contempt, domestic violence [51,52]. Cognitive/behavioural skill training exercises and role plays use the experiences of the participants in problem solving and cognitive restructuring, and participants are guided in applying cognitive restructuring skills to practicing safer sex and improving communication [53]. At the close of the session, participants receive a weeks's supply of their most preferred products male and female condoms.

*Session Four *begins with participants' completion of sexual diaries, sexual barrier attitudes and behaviours, and psychosocial questionnaires. This session focuses on conflict resolution [54] communication and sexual negotiation within the relationship. Videos and role playing are used to illustrate elements of open successful communication and elements of negative communication, such as negativity, escalation and invalidation. Participants are trained in communication with empathy, increased positive messages and the reduction of negative verbal messages. At the close of the session, participants receive a month's supply of their most preferred products. Participants will be advised that they can come to the study site to receive up to one month's supply of barrier products each month for the duration of their participation in the study.

#### Counsellor training and intervention quality assurance

Sites selected will have identified characteristics needed for successful implementation, including patient volume, space, staff and potential for sustainability [55]. These characteristics will be reviewed prior to study onset, and ongoing staff clinic meetings will be used to assess and respond to challenges as they arise.

Study personnel will undergo formal training in recruitment, assessment, and intervention procedures. Training includes in-depth review of all assessment measures, intensive review of the intervention manual, the PMTCT protocol, and training in cognitive behavioural intervention strategies used in the intervention. Intervention and assessment fidelity will be maintained by tape recording of intervention sessions and quality control will be conducted with transcribed sessions.

### Outcome Measures

**Assessments Months 3-10**. All study materials, e.g., consent, assessment and intervention, will be translated into the major local languages (Zulu, Swati, Ndebele). Both participants and clinic sites will be assessed (see Assessment Table 1). Participant assessments will include both biological (clinic data) and psychosocial assessments at study entry, post-PartnerPlus intervention, 32 weeks prior to delivery, immediately prior to delivery, 3 days post-delivery and 6 weeks post-delivery. All assessments are based on barriers to PMTCT uptake and will be interviewer administered to avoid exclusion based on literacy. Participants will receive R100 per assessment and R50 per intervention session for time and transportation.

**Table 1:** Assessment Measures: Individual Participants [Maternal, Paternal, Infant]

Assessment	Time (mins)	PMTCT Protocol	Pre-enrolment Screen	Base-line	Post- Interven-tion	32 wks pre delivery	Deliv-ery	3 days Post- Delivery	6 weeks post- Delivery
**PMTCT Standard of Care Protocol**									

HCT maternal	**20**	**X**							
Maternal CD4	**5**	**X**							
ART	**30**	**X**							
ARV prophylaxis	**5**	**X**							**X**
Paternal HCT	**20**	**X**							
Paternal CD4		**X**							
Maternal 32 wk. HCT	**20**	**X**				**X**	**X**		
PCR - infant - HIV	**5**	**X**							**X**

**Screening**									

Couples Verification	**10**		**X**						

**Biological Assessment**									
Maternal & infant ART & ARV prophylaxis blood test	**5**							**X**	

**Psychosocial & Behavioural Assessment**									

Demographics	**5**			**X**	**X**				
PMTCT& HIV Knowledge	**5**			**X**	**X**				
Adherence: 4 days ART & ARV prophylaxis	**5**			**X**	**X**				
Sexual diary: 7 days	**5**			**X**	**X**				
Sexual Risk Behaviour: 1 month	**10**			**X**	**X**				
Stigma	**10**			**X**	**X**				
Intimate Partner Violence: 1 month, lifetime	**5**			**X**	**X**				

### Biological Assessment

#### Clinic data

Patient record data on the standard of care for PMTCT and HCT, including, 1) baseline HCT, HCT at 32 weeks and HCT pre-delivery for women (standard of care, PMTCT), 2) HCT for male partners (standard of care, HCT) and 3) HIV testing by PCR at 6 weeks for infants (standard of care, PMTCT).

#### ARV uptake assessment

Maternal and infant blood samples will be collected within 7 days post- delivery using dried blood spots (DBS). Samples will be analyzed to obtain a quantitative assessment of the presence of Nevirapine, Zidovudine, Efavirenz, Lamivudine, Stavudine, Lopinavir, Tenofovir and Emtricitabine, the current medication regimens for HAART and PMTCT in South Africa.

### Assessment of Site Characteristics & Participants: Maternal, paternal & infant

**Site**: Organizational Characteristics for PMTCT services [55].

**Participants**: Session attendance, PMTCT visit attendance, HIV testing.

Demographics (includes family composition, serostatus disclosure, HIV status).

HIV and PMTCT Knowledge.

Adherence (Self reported ARV uptake - 4 days (ACTG 4-day Adherence Self Report measure) [56].

Sexual Risk (Sexual Risk Behaviour Scale) [57] Sexual Barrier Use (Sexual Diary).

Stigma (Stigma Indicators Scale).

Intimate Partner Violence (Conflict Tactics Scale) [58].

### Analysis approach

#### Statistical analysis

The goal of this study is to combine an effective sexual risk reduction intervention with an effective medication adherence intervention to increase adherence to the PMTCT protocol. Clustering has been used due to the anticipated observed intercorrelations within the clinics. The sample size has been estimated as 1 + (m-1) p, m being the average cluster size and p being the intracluster correlation coefficient. All comparisons will use an alpha (two-tailed) of 0.05. Baseline characteristics will be described using medians and interquartile ranges for continuous variables and percentages with binomial confidence intervals for categorical variables. This is a group-randomized study that randomly assigns 12 sites with 10 couples per site to either the intervention arm or the control arm in a 1:1 ratio.

##### Hypothesis 1

Community clinics implementing PartnerPlus will have more effective PMTCT program adherence, as measured by maternal and paternal ANC and PartnerPlus visits, maternal and paternal HCT uptake, maternal and infant Nevirapine and AZT dose uptake, infant PCR, maternal and infant HIV serostatus.

##### Hypothesis 1a

Community clinics implementing PartnerPlus will have reduced sexual risk behaviour, as measured by participant sexual barrier use.

##### Analytic Plan 1 & 1a

We will use a general estimating equations (GEE) model to perform a Poisson regression to test whether the likelihood of ANC attendance is significantly higher in the intervention arm than the control arm adjusting for the clustering effect (Primary Hypothesis). Assuming that 80% of the men in the intervention arm and 2% of the men in the control arm attend ANC and a two-tailed test at the 0.05 level, we estimate the power to be 0.99 for a range of intracluster correlation coefficients from 0.1 to 0.9 [59]. A linear mixed model will be used to compute a repeated measures analysis of variance to compare pre and post sexual risk scores between the intervention and control groups adjusting for cluster effects (Secondary Hypothesis). Contrasts will be used to test planned comparisons of pre and post scores between groups and pre-post change within and between groups.

#### Laboratory Methods

##### Specimen tracking

Batches of specimens will be sent to a laboratory at the University of Cape Town and will be tracked through waybill procedures. Specimens and specimen tracking sheets with the Dry Blood Spots (DBS) bar-code will be sent to the laboratory in transparent zipper lock bags containing desiccant. Consecutively numbered laboratory bar-codes will be assigned to the specimens as they are received by the laboratory. The specimen bar-codes will by matched to the bar-codes on the laboratory tracking sheets. The specimen bar-code number will also be scanned or typed into an excel spreadsheet. The Guthrie cards will also be labelled with the laboratory bar-code number. Laboratory managers will perform a second quality control (matching bar-codes to tracking sheets and examining specimen quality) and will sign-off the tracking sheets for laboratory processing.

#### Detection of antiretroviral drugs

The presence of antiretroviral drugs in HIV positive DBS samples will be confirmed by means of High Performance Liquid Chromatography coupled to Tandem Mass Spectrometry. Qualitative detection of Nevirapine, Zidovudine, Efavirenz, Lamivudine, Stavudine, Lopinavir, Tenofovir and Emtricitabine in DBS samples will be carried out by a validated method using minor modifications of the method used by [60].

#### Ethical and research governance approval

We have received ethical approval from the Human Sciences Research Council Research Ethics Committee (Protocol REC No. 1/18/08/10), the University of Miami Miller School of Medicine (Protocol No. 20100555), the National Institute of Allergies and Infectious Diseases and the Provincial Department of Health of Mpumalanga, South Africa.

#### Project Timescales

The study will run for a period of 13 months beginning in December 2010 to December 2011.

## Discussion

Shared intervention elements may decrease sexual risk and enhance PMTCT uptake, e.g., increased male participation, enhanced communication, HCT, adherence, serostatus disclosure, suggest that a combined sexual risk reduction and adherence intervention *plus *PMTCT can increase male participation, increase couples' communication and encourage adherence to the PMTCT process. This intervention presents a method to implement a couples-based PMTCT intervention that simultaneously decreases sexual risk and increases adherence to PMTCT uptake by increasing male involvement. The intervention, PartnerPlus, combines an evidence-based behavioural HIV risk reduction intervention, the Partner Project, with an effective pharmacologic adherence intervention to prevent mother to child transmission, PMTCT. The findings will impact public health and will enable the health ministry to formulate policy related to male involvement in PMTCT, which will result in PMTCT.

## List of abbreviations used

ACTG: AIDS Clinical Trial Group; ANC: Antenatal Care; ARV: antiretroviral drugs; ART: Antiretroviral Therapy; AZT: Azidothymidine or Zidovudine; DBS: Dry Blood Spots; PCR: Polymerase chain reaction; PMTCT: Prevention of HIV Transmission from Mother to Child; HCT: HIV Counselling and Testing; STI: Sexually Transmitted Infection.

## Competing interests

The authors declare that they have no competing interests.

## Authors' contributions

KP, DJ and SW were the main contributors to the conceptualization of the study. KP and DJ also contributed significantly to the first draft of the paper and all authors contributed to the subsequent drafts and finalization. All authors read and approved the final manuscript.

## Pre-publication history

The pre-publication history for this paper can be accessed here:

http://www.biomedcentral.com/1471-2458/11/778/prepub

## References

[B1] UNAIDS Declaration of commitment on HIV/AIDS. United Nations General Assembly: 495 Special Session on HIV/AIDS 2001 United Nations, New York, New York

[B2] Department of Health National antenatal sentinel HIV and syphilis prevalence survey 2009 Pretoria: Department of Health http://www.info.gov.za/view/DownloadFileAction?id=109007 retrieved 20 April 2011

[B3] Doherty T Besser M Donohue S Kamoga N Stoops N Williamson L Visser R An evaluation of the Prevention of Mother-to-child Transmission (PMTCT) of HIV initiative in South Africa: lessons and key recommendations 2003 Durban: Health Systems Trust

[B4] Shisana O Simbayi LC Rehle T Zungu NP Zuma K Ngogo N Jooste S Pillay-Van Wyk V Parker W Pezi S Davids A Nwanyanwu O Dinh TH SABSSM III Implementation Team South African national HIV prevalence, HIV incidence, behaviour and communication survey, 2008: The health of our children 2010 Cape Town: Human Sciences Research Council Press

[B5] Rispel LC Peltzer K Phaswana-Mafuya N Metcalf CA Treger L Assessing missed opportunities for the prevention of mother-to-child HIV transmission (PMTCT) in the Kouga Local Service Area (LSA), Eastern Cape S Afr Med J 2009 99 3 174 179 19563095

[B6] Peltzer K Phaswana-Mafuya N Ladzani R Implementation of the national programme for prevention of mother-to-child transmission of HIV: A rapid assessment in Cacadu district, South Africa Afr J AIDS Res 2010 9 1 95 106 10.2989/16085906.2010.484594 25860417

[B7] Expert Panel Report: Prevention of Mother-to-Child Transmission of HIV: Expert Panel Report and Recommendations to the U.S. Congress and U.S. Global AIDS Coordinator January 2010 http://www.pepfar.gov/documents/organization/135465.pdf retrieved 20 April 2011

[B8] Moodley D Esterhuizen TM Pather T Chetty V Ngaleka L High HIV incidence during pregnancy: Compelling reason for repeat HIV testing AIDS 2009 23 10 1255 1259 http://www.medscape.com/medline/abstract/19455017 10.1097/QAD.0b013e32832a5934 19455017

[B9] Kinuthia J Kiarie JN Farquhar C Richardson B Nduati R Mbori-Ngacha D John-Stewart G Cofactors for HIV-1 incidence during pregnancy and postpartum period Curr HIV Res 2010 8 7 510 4 10.2174/157016210793499213 20946093 PMC3372399

[B10] Theuring S Mbezi P Luvanda H Jordan-Harder B Kunz A Harms G Male involvement in PMTCT services in Mbeya Region, Tanzania AIDS Behav 2009 13 S1 92 102 10.1007/s10461-009-9543-0 19308720

[B11] Joseph D Projet San Francisco Rwanda-Zambia HIV Research Group Emory University Rollins School of Public Health Kigali, Rwanda Improving on a Successful Model for Promoting Couples' VCT in Two African Capitals: Mobile Couples' HIV Testing Units Proceedings of XV International AIDS Conference 2004 Bangkok, Thailand

[B12] UNAIDS Report on the global HIV/AIDS epidemic http://www.unaids.org/en/KnowledgeCentre/HIVData/GlobalReport/2008/2008_Global_report.asp. Accessed 25 May 2010

[B13] Stringer JS Sinkala M Maclean CC Levy J Kankasa C Degroot A Stringer EM Acosta EP Goldenberg RL Vermund SH Effectiveness of a city-wide program to prevent mother-to-child HIV transmission in Lusaka, Zambia AIDS 2005 19 12 1309 15 10.1097/01.aids.0000180102.88511.7d 16052086 PMC2745046

[B14] Stringer EM Chi BH Namwinga C Creek TL Ekouevi DK Coetzee D Stringer JSA Monitoring effectiveness of programs to prevent mother-to-child transmission in lower-income countries Bull World Health Organ 2008 86 1 1 80 10.2471/BLT.07.043117 PMC2647351 18235891

[B15] Kieffer MP Hoffman H Nlabhatsi B Mahdi M Kudiabor K Wilfert C Lukhele V Nakato-Waligo A Maseko N Repeat HIV testing in labor and delivery as a standard of care increases ARV provision for women who seroconvert during pregnancy (abstract no. 156) Proceedings of the 17th Annual Conference on Retroviruses and Opportunistic Infections (CRO) 2010 San Francisco, C.A

[B16] Peltzer K Mlambo G Phaweni K Factors determining prenatal HIV testing for prevention of mother to child transmission of HIV in Mpumalanga, South Africa AIDS Behav 2010 14 5 1115 23 10.1007/s10461-009-9662-7 20049520

[B17] Peltzer K Chao LW Dana P Family planning among HIV positive and negative Prevention of Mother to Child (PMTCT) clients in a resource poor setting in South Africa AIDS Behav 2009 13 5 973 9 10.1007/s10461-008-9365-5 18286365

[B18] Kuonza LR Tshuma CD Shambira GN Tshimanga M Non-adherence to the single dose nevirapine regimen for the prevention of mother-to-child transmission of HIV in Bindura town, Zimbabwe: a cross-sectional analytic study BMC Public Health 2010 10 1 218 10.1186/1471-2458-10-218 20426830 PMC2873585

[B19] Mbonye AK Hansen KS Wamono F Magnussen P Barriers to prevention of mother-to-child transmission of HIV services in Uganda J Biosoc Sci 2009 9 1 13 10.1017/S002193200999040X 19895727

[B20] Peltzer K Mosala T Dana P Fomundam H Follow-up survey of women who have undergone Prevention of Mother to Child Transmission (PMTCT) in a resource poor setting in South Africa JANAC 2008 19 6 450 460 10.1016/j.jana.2008.05.006 19007723

[B21] Peltzer K Mlambo M Phaswana-Mafuya N Ladzani R Determinants of adherence to a single-dose nevirapine regimen for the prevention of mother-to-child HIV transmission in Gert Sibande district in South Africa Acta Paediatrica 2010 99 5 699 704 20146724 10.1111/j.1651-2227.2010.01699.x

[B22] Desgrées-du-Loû A Orne-Gliemann J Couple-centred testing and counselling for HIV serodiscordant heterosexual couples in sub-Saharan Africa Reprod Health Matters 2008 16 32 151 61 10.1016/S0968-8080(08)32407-0 19027631

[B23] Becker S Mlay R Schwandt HM Lyamuya E Comparing Couples' and Individual Voluntary Counseling and Testing for HIV at Antenatal Clinics in Tanzania: A Randomized Trial AIDS Behav 2009 14 3 558 66 19763813 10.1007/s10461-009-9607-1

[B24] African Development Bank The development of harmonized minimum standards for guidance on HIV testing and counseling and prevention of mother-to-child transmission of HIV in the SADC Region. PMTCT Country Report Lesotho 2009 http://www.hsrc.ac.za/research/output/outputDocuments/6312_Agu_PMTCT_Lesotho.pdf Retrieved on 20 April 2011

[B25] Desgrées-du-Loû A Brou H Traore AT Djohan G Becquet R Leroy V From prenatal HIV testing of the mother to prevention of sexual HIV transmission within the couple Soc Sci Med 2009 69 6 892 9 10.1016/j.socscimed.2009.05.045 19552991

[B26] Farguhar C James K Barbra R Marjory K Francis J Ruth N Dorothy M Grace S Antenatal couple counseling increases uptake of interventions to prevent HIV-1 transmission J Acquir Immune Defic Syndr 2004 37 5 1620 6 10.1097/00126334-200412150-00016 15577420 PMC3384734

[B27] Katz DA Kiarie JN John-Stewart GC Richardson BA John FN Farquhar C Male perspectives on incorporating men into antenatal HIV counseling and testing PLoS One 2009 4 11 e7602 10.1371/journal.pone.0007602 19881884 PMC2765726

[B28] Katz DA Kiarie JN John-Stewart GC Richardson BA John FN Farquhar C HIV testing men in the antenatal setting: Understanding male non-disclosure Int J STD AIDS 2009 20 11 765 7 10.1258/ijsa.2009.009139 19833691 PMC3042848

[B29] Allen S Conkling M Shutes EL Karita E Chomba E Tichacek A Sinkala M Vwalika B Iwanowski M Allen SA Couples' voluntary counselling and testing and nevirapine use in antenatal clinics in two African capitals: a prospective cohort study J Int AIDS Soc 2010 15;13 1 10 20230628 10.1186/1758-2652-13-10 PMC2851580

[B30] Orne-Gliemann J Tchendjou PT Miric M Gadgil M Butsashvili M Eboko F Perez-Then E Darak S Kulkarni S Kamkamidze G Balestre E Desgrees du Lou A Dabis F Couple-oriented prenatal HIV counseling for HIV primary prevention: an acceptability study BMC Public Health 2010 10 197 10.1186/1471-2458-10-197 20403152 PMC2873579

[B31] Auvinen J Suominen T Välimäki M Male participation and prevention of human immunodeficiency virus (HIV) mother-to-child transmission in Africa Psychol Health Med 2010 15 3 288 313 10.1080/13548501003615290 20480434

[B32] Simbayi LC Kalichman SC Condom failure in South Africa S Afr Med J 2007 97 7 476 17805445

[B33] Kalichman SC Rompa D Cage M Group intervention to reduce HIV transmission risk behavior among persons Behavior Modification 2005 29 2 256 85 10.1177/0145445504272603 15657411

[B34] Bunnell RE Nassozi J Marum E Mubangizi J Malamba S Dillon B Kalule J Bahizi J Musoke N Mermin JH Living with discordance: knowledge, challenges, and prevention strategies of HIV-discordant couples in Uganda AIDS Care 2005 17 8 999 1012 10.1080/09540120500100718 16176896

[B35] Chen YQ Young A Brown ER Chasela CS Fiscus SA Hoffman IF Valentine M Emel L Taha TE Goldenberg RL Read JS Population attributable fractions for late postnatal mother-to-child transmission of HIV-1 in Sub-Saharan Africa J Acquir Immune Defic Syndr 2010 54 3 311 6 10.1097/QAI.0b013e3181d61c2e 20224418 PMC3086731

[B36] Jones D Ross D Weiss SM Bhat G Chitalu N Influence of partner participation on sexual risk behavior reduction among HIV-positive Zambian women J Urban Health 2005 82 92 100 10.1093/jurban/jti111 16107445

[B37] Jones D Weiss SM Bhat GJ Feldman SA Bwalya V Budash D A sexual barrier intervention for HIV+/- Zambian women: acceptability and use of vaginal chemical barriers J Multicult Nurs Health 2004 10 27 31 PMC3034147 21304832

[B38] Jones DL Bhat GJ Weiss SM Feldman DA Bwalya V Influencing sexual practices among HIV positive Zambian women AIDS Care 2006 18 629 635 10.1080/09540120500415371 16831792 PMC2483952

[B39] Jones DJ Chitalu N Ndubani P Mumbi M Weiss SM Villar-Loubet O Vamos S Waldrop-Valverde D Sexual risk reduction among Zambian couples SAHARA J 2009 6 2 69 75 19936408 10.1080/17290376.2009.9724932 PMC3731986

[B40] Department of Health HIV and AIDS and STI strategic plan for South Africa, 2007-2011 2007 Pretoria: Department of Health

[B41] Day C Barron P Monticelli F Sello E The district health barometer - year 2007/08 (Technical Report) 2009 Durban: Health Systems Trust http://www.hst.org.za/publications/850 retrieved on 20 April 2011

[B42] McMahon JM Tortu S Torres L Pouget ER Hamid R Recruitment of heterosexual couples in public health research: a study protocol BMC Med Res Methodol 2003 31;3 24 14594457 10.1186/1471-2288-3-24 PMC272932

[B43] Ajzen I Fishbein M Understanding attitudes and predicting social behavior 1980 Englewood Cliffs, NJ: Prentice-Hall

[B44] Ajzen I From intentions to actions: A theory of planned behavior. In J Kuhi, J Beckmann (Eds.) Action control: From cognition to behavior 1985 Heidelberg: Springer 11 39

[B45] Albarracín D Johnson BT Fishbein M Muellerleile PA Theories of reasoned action and planned behavior as models of condom use: A meta-analysis CHIP Documents. Paper 8, 2001. http://digitalcommons.uconn.edu/chip_docs/8, Retrieved on 20 April 2011 10.1037/0033-2909.127.1.142 PMC4780418 11271752

[B46] Stein Z More on women and the prevention of HIV infection (Editorial) Am J Public Health 1995 85 1485 8 10.2105/AJPH.85.11.1485 7485657 PMC1615705

[B47] Gollub EL French P Latka M Rogers C Stein Z Achieving safer sex with choice: studying a women's sexual risk reduction hierarchy in an STD clinic J Womens Health Gend Based Med 2001 10 8 771 83 10.1089/15246090152636532 11703890

[B48] Baucom DH Epstein N Cognitive behavioral marital therapy 1990 New York: Brunner/Mazel

[B49] Quina K Harlow LL Marokoff PJ Burkholder G Deiter PJ Sexual communication in relationships: When words speak louder than actions Sex Roles 2000 42 523 549 10.1023/A:1007043205155

[B50] Noar SM Morokoff PJ Harlow LL Condom negotiation in heterosexually active men and women: Development and validation of a condom influence strategy questionnaire Psychology and Health 2002 17 711 735 10.1080/0887044021000030580

[B51] Frei JR Shaver PR Respect in close relationships: Prototype definition, self-report assessment, and initial correlates Pers Relationships 2002 9 121 139 10.1111/1475-6811.00008

[B52] Gottman JM The roles of conflict engagement, escalation, and avoidance in marital interaction: A longitudinal view of five types of couples J Consult Clin Psychol 1993 61 1 6 15 8450108 10.1037//0022-006x.61.1.6

[B53] Sillars A Roberts LJ Leonard KE Dun T Cognition during marital conflict: The relationship of thought and talk J Soc Pers Relationships 2000 17 479 502 10.1177/0265407500174002

[B54] Glasgow ER Translating research to practice Diabetes Care 2003 26 8 2451 6 10.2337/diacare.26.8.2451 12882877

[B55] Family Health International Baseline Assessment Tools for Preventing Mother-to-Child Transmission (PMTCT) of HIV 2003 Arlington, Virginia, USA: Institute for HIV/AIDS

[B56] Chesney MA Ickovics JR Chambers DB Gifford AL Neidig J Zwickl B Wu AW Self-reported adherence to antiretroviral medications among participants in HIV clinical trials: the AACTG adherence instruments. Patient Care Committee & Adherence Working Group of the Outcomes Committee of the Adult AIDS Clinical Trials Group (AACTG) AIDS Care 2000 12 3 255 266 10.1080/09540120050042891 10928201

[B57] Meyer-Bahlberg HFL Ehrhardt AA Exner TM Gruen RS Sexual Risk Behavior Assessment Schedule: Adult. (SERBAS-A-DF-4) Manual 1990 New York: Psychological Press

[B58] Strauss MA Hamby SL Boney-McCoy S Sugarman DB The Revised Conflict Tactics Scales (CTS2) Journal of Family Issues 1996 17 3 283 316 10.1177/019251396017003001

[B59] Donner A Klar N Design and analysis of cluster randomization trials 2000 London: Arnold 10.1002/sim.1115 11782029

[B60] Koal T Burhenne H Romling R Svoboda M Resch K Quantification of antiretroviral drugs in dried blood spot samples by means of liquid chromatography/tandem mass spectrometry Rapid Communications in Mass Spectrometry 2005 19 2995 3001 10.1002/rcm.2158 16193530

